# Rare thyroid non-neoplastic diseases

**DOI:** 10.1186/s13044-015-0017-3

**Published:** 2015-04-11

**Authors:** Katarzyna Lacka, Adam Maciejewski

**Affiliations:** Department of Endocrinology, Metabolism and Internal Medicine, University of Medical Sciences, Poznan, Poland

**Keywords:** Rare disease, Thyroid gland, Congenital hypothyroidism, Thyroxin binding globulin, Transthyretin, Thyroid hormone resistance, Dysgenesis, Dyshormonogenesis, Mutation

## Abstract

Rare diseases are usually defined as entities affecting less than 1 person per 2,000. About 7,000 different rare entities are distinguished and, among them, rare diseases of the thyroid gland. Although not frequent, they can be found in the everyday practice of endocrinologists and should be considered in differential diagnosis. Rare non-neoplastic thyroid diseases will be discussed. Congenital hypothyroidism’s frequency is relatively high and its early treatment is of vital importance for neonatal psychomotor development; CH is caused primarily by thyroid dysgenesis (85%) or dyshormonogenesis (10-15%), although secondary defects - hypothalamic and pituitary - can also be found; up to 40% of cases diagnosed on neonatal screening are transient. Inherited abnormalities of thyroid hormone binding proteins (TBG, TBP and albumin) include alterations in their concentration or affinity for iodothyronines, this leads to laboratory test abnormalities, although usually with normal free hormones and clinical euthyroidism. Thyroid hormone resistance is most commonly found in *THRB* gene mutations and more rarely in *THRA* mutations; in some cases both genes are unchanged (non-TR RTH). Recently the term ‘reduced sensitivity to thyroid hormones’ was introduced, which encompass not only iodothyronine receptor defects but also their defective transmembrane transport or metabolism. Rare causes of hyperthyroidism are: activating mutations in *TSHR* or *GNAS* genes, pituitary adenomas, differentiated thyroid cancer or gestational trophoblastic disease; congenital hyperthyroidism cases are also seen, although less frequently than CH. Like other organs and tissues, the thyroid can be affected by different inflammatory and infectious processes, including tuberculosis and sarcoidosis. In most of the rare thyroid diseases genetic factors play a key role, many of them can be classified as monogenic disorders. Although there are still some limitations, progress has been made in our understanding of rare thyroid diseases etiopathogenesis, and, thanks to these studies, also in our understanding of how normal thyroid gland functions.

## Introduction

Rare diseases are usually defined as entities affecting 5 or fewer per 10,000 (1 person per 2,000), although different thresholds can also be found, e.g. in Japan - fewer than 4 cases per 10,000 or in the United States - fewer than 200,000 patients affected across the country. Despite this rarity, the total number of a people with diagnosis of rare diseases is estimated at 350 million all over the world. Approximately 7,000 distinct rare entities are described, most of them genetically determined [1]. Another term - ultra-rare diseases - is usually referred to diseases that affect less than 1 person per 50,000 [2].

On the basis of prevalence, some thyroid gland diseases can also be classified as rare. They have genetic origin in most cases, often as a result of single gene mutations (monogenic disorders). Rare thyroid diseases should be considered in differential diagnosis of commonly seen thyroid entities, their right diagnosis may be often of vital importance. The aim of this review is therefore to briefly present the spectrum of rare thyroid diseases and underling etiopathogenesis. Rare thyroid diseases can be classified into two main categories: neoplastic and non-neoplastic disorders. Rare thyroid non-neoplastic entities will be discussed further in this paper.

## Review

### Congenital hypothyroidism

Congenital hypothyroidism (CH) can be classified as primary or secondary. The causes of primary CH are thyroid dysgenesis or dyshormonogenesis. Secondary CH may be: 1) hypothalamic (also called tertiary CH) – *TRH gene* mutations; 2) pituitary – congenital hypopituitarism with multihormonal insufficiency (some known mutations of transcription factors’ genes such as *Pit 1, Prop 1, LHX 3, LHX 4, HESX1*, but 80-90% of idiopathic origin) or isolated TSH deficiency (*TSHB* or *rTRH genes* mutations). Some cases of congenital thyroid hormones deficiency are transient (the percentage varies between studies, up to 40%) and euthyroidism is achieved within the first months or years of life (maternal antithyroid drug intake, maternal TSHR blocking antibodies, iodine deficiency or excess, some heterozygous mutations of DUOX2 and DUOXA2, congenital liver hemangioma) [3]. Lack of thyroid hormone action at birth may also result from peripheral causes (peripheral congenital hypothyroidism), which will be discussed in subsequent paragraphs.

**Figure 1:**
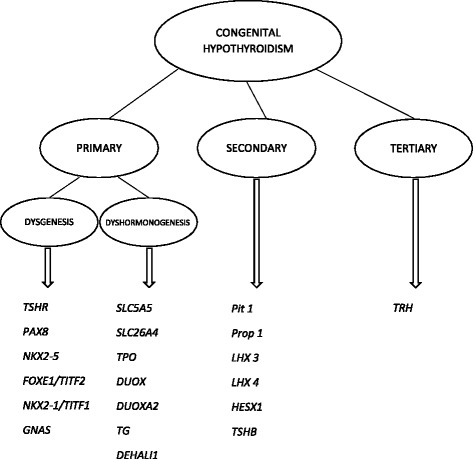
**Genetic background of congenital hypothyroidism.**

The general prevalence of CH, regardless of its etiology, is 1:2,000 to 1:4,000 of neonates (primary CH - 1:4,000, secondary CH – 1:66,000) with a female-to-male ratio of 2:1. These values vary between different regions (1:800 in the Greek Cypriot population, 1:2,000 in China, 1:2,300 in the US and 1:10,000 in France) or ethnic groups (highest prevalence in Asians) [3-5]. In Poland it is estimated that CH affects about 1:4,500 of all screened neonates [6]. In iodine sufficient regions, the vast majority of CH cases are caused by thyroid dysgenesis (up to 85%) and dyshormonogenesis (10-15%), while secondary CH is responsible for only 0.0015% of all cases [7]. The genes involved in CH etiopathogenesis are presented in Figure 1.

#### Dysgenesis

The term thyroid dysgenesis includes the ectopic location of the gland (thyroid ectopy), absence (athyreosis) and underdevelopment of the thyroid tissue (hypoplasia). Unilateral thyroid agenesis (hemiagenesis) is also classified as thyroid dysgenesis, although usually with clinical euthyroidism [8]. Within the group of thyroid dysgenesis, ectopy is found most commonly, with lingual, supra- and infrahyoid localization of the gland most frequently seen. Cases of thyroid dysgenesis are usually sporadic, inherited genetic defects are only recognized in about 2% of all cases and result from transcription factor genes mutations (*PAX8, NKX2-1/TITF1, NXK2-5, FOXE1/TITF-2*) [9]. As these transcription factors are also involved in extrathyroid development, different organs’ congenital anomalies may coexist (Table 1). The thyroid gland is the only organ affected by *TSHR gene* (14q31.1) mutations, which account for about 1% of all CH cases. As more than 60 different *TSHR* loss-of-function mutations have been found (Y444X described for the first time in the Polish population), a variable degree of TSH resistance and ongoing clinical presentation are observed (from euthyroid hyperthyrotropinemia to hypoplasia with severe hypothyroidism) [10,11]. TSH resistance is also observed in mutations of *GNAS gene* (20q13.32), encoding the alpha subunit of G protein, downstream of TSH receptor in the signaling pathway. As G protein is also responsible for signal transduction of other peptide hormones, in these cases resistance is multihormonal and the clinical picture is complex (pseudohypoparathyroidism type 1a) [12]. The role of environmental factors (e.g. intrauterine viral infections) in thyroid dysgenesis is also suggested [13].

**Table 1:** **Transcription factor genes responsible for thyroid dysgenesis**

**Gene**	**Locus**	**Type of inheritance**	**Expression**	**Clinical manifestation**
*PAX8*	2q13	AD	Thyroid, kidney, CNS	Thyroid hypoplasia or athyreosis, agenesis/hemiagenesis of the kidneys
*NKX2-5*	5q35.1	AD	Thyroid, heart, pharynx	Thyroid ectopy, athyreosis, congenital cardiac malformations
FOXE1/TITF2	9q22.33	AR	Thyroid, pituitar, tongue, epiglottis, palate, pharynx, thymus and Rathke’s pouch, choanae, hair follicles	Athyreosis, other forms of thyroid dysgenesis, cleft palate, cleft epiglottis, choanal atresia, spiky hair
*NKX2-1/TITF1*	14q13.3	AD	Thyroid, lung, trachea, CNS	Thyroid dysgenesis, dyshormonogenesis due to inhibited *TG* expression, choreoatetosis, hypotony, respiratory distress

#### Dyshormonogenesis

**Figure 2:**
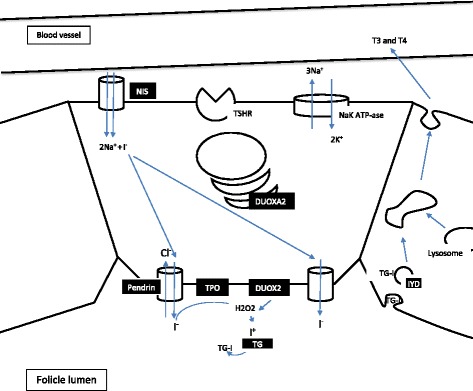
**Thyroid hormone biosynthesis (possible genetic defect sites shown in black).**

Another reason for primary CH – dyshormonogenesis – arises from defective thyroid hormone synthesis; all its stages may be affected (Figure 2). These monogenic defects are inherited predominantly with an autosomal recessive pattern. Clinically, in addition to the symptoms and signs of thyroid hormone deficiency (rarely euthyroidism), patients may present with goiter.

Mutations in the *SLC5A5 gene* (19p13.11) encoding sodium-iodide symporter (NIS) result in a defect in the active transport of iodide to thyrocytes. NIS glycoprotein is expressed not only in the thyroid follicular cells, but also in some non-thyroid tissues – salivary and lacrimal glands, cancerous breast tissue, the breast during lactation, the intestines, stomach, testes and placenta. To data, at least 12 different loss-of-function mutations in the *NIS gene* have been described [14]. This relatively rare cause of dyshormonogenesis is characterized by an autosomal recessive pattern of inheritance. Diminished or completely inhibited iodine uptake by the thyroid is characteristic.

Pendrin, encoded by *SLC26A4 gene* (7q22.3)*,* is an anion transporter expressed primarily in the thyroid (allows iodide efflux into the follicular lumen) but also in the inner ear, kidneys and lungs. Bi-allelic mutations of the *SLC26A4 gene* (about 470 mutations described to date) lead mainly to two different phenotypes: a) non-syndromic hearing loos with enlarged vestibular aqueduct (EVA) or b) Pendred syndrome (PDS) with congenital bilateral sensorineural hearing loss and thyroid defect (diffuse or multinodular goiter, thyroid hormone status is dependent on iodide intake, with dominance of euthyroidism in iodide sufficient regions) [15-17]. PDS is a rare cause of CH (2-3%), goiter often occurs later, in childhood or early adolescence. Patients with PDS are at increased risk of thyroid cancer development (about 1%), most likely follicular carcinoma [18].

Thyroid peroxidase (TPO) catalyze iodide oxidation, tyrosyl residue iodination and their coupling to iodothyronines [19]. *TPO* (2p25.3) mutation is considered to be the most prevalent cause of dyshormonogenesis (24-46%), and is found in about 1:60,000 newborns, with about 90 different mutations already described [15,20]. The disease is generally inherited with an autosomal recessive manner, although monoallelic mutations may be a risk factor for transient hypothyroidism.

In the process of iodothyronine synthesis, H2O2 is required, which is produced by the dual oxidase enzyme (DUOX/ThOX). Two variants of DUOX can be distinguished, with type 2 being critical for thyroid hormone synthesis [21]. In the case of *DUOX2* (15q21.1), autosomal dominant mode of inheritance is observed, although homozygotes are usually affected more severely. Patients with mono-allelic mutations typically present with transient congenital hypothyroidism, but should be monitored in adulthood in situations characterized by an increased thyroid hormone requirement, such as pregnancy [22]. Defects in another thyroid protein - *DUOXA2* (15q21.1), which is indispensable for DUOX2 maturation and translocation, can also cause CH [23].

Thyroglobulin (TG) is essential in thyroid hormone synthesis as it provides tyrosyl residues and enable the storage of hormones and iodine. Since it was first described, more than 50 different mutations that lead to CH have been found [24]. *TG* (8q24.22) mutations are described as one of the most prevalent causes of dyshormonogenesis ( 1:67,000 to 1:100,000) [24,25]. A characteristically undetectable or very low TG level is observed in these patients, T4 concentration is found to be disproportionately lower than T3 (a result of increased type-2 iodothyronine deiodinase activity) [26].

Although the end products of hormonogenesis are T3 and T4, most iodine particles are embedded to mono- and diiodotyronines (MIT, DIT). Reuse of this iodine is possible due to MIT and DIT deiodination by an iodotyrosine deiodinase (IYD). Mutations in the *IYD gene* (6q25.1) may lead to goitrous hypothyroidism [27]. A characteristic feature is excessive urinary and blood MIT and DIT concentrations [28].

### Inherited thyroid hormone binding protein abnormalities

#### TBG

Thyroxin binding globulin (TBG) is the main protein that binds T3 and T4 in the blood (70% and 70-80% respectively); other transporting particles (transthyretin and albumin) play a minor role. TBG is encoded by the *Serpina 7 gene* (Xq22.3) and expressed in the liver [29]. Three different congenital TBG abnormalities are distinguished according to its concentration: complete deficiency (TBG-CD), partial deficiency (TBG-PD) and excess (TBG-E). They are inherited in a X-linked recessive pattern (although a case of autosomal dominant inheritance of TBG-PD has been described) [30]. There are also some *TBG* polymorphic variants that do not alter the protein concentration.

**Figure 3:**
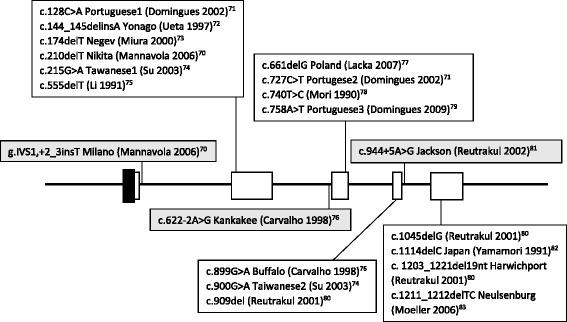
**Mutations responsible for TBG-CD (mutations in coding regions shown in white boxes, mutations in non-coding regions shown in grey boxes) [** 70-83 **].**

TBG-CD is diagnosed when the protein level is undetectable by typically used assays (or is lower than 0.03% of the normal mean value), and can be found in hemizygous men and homozygous women. Heterozygous women usually have only a slightly diminished TBG concentration (carriers), although rarely, as a result of selective X chromosome inactivation, TBG-CD may develop [31]. Men with TBG-PD have a significantly decreased serum concentration of globulin, while women are found to have values that may even overlap the normal range. TBG deficiency is estimated to occur in 1:15,000 to 1:5,000 of all newborns in the Caucasian population (there is a higher frequency in the Japanese population), with TBG-CD in one-third of them [32]. At least 22 different TBG-CD and 8 TBG-PD mutations have been described (TBG-CD mutations shown on Figure 3) [33]. Laboratory tests in these generally euthyroid patients show normal TSH, fT3 and fT4 with a low T4, T3 and undetectable TBG level.

Excessive TBG concentration is typically observed in the course of elevated estrogen level (e.g. pregnancy). Congenital TBG-E occurs rarely, as a consequence of gene duplication or triplication (in 1:6,000 to 1:40,000 of all newborns) [34]. In such cases, the values measured are 2–3 times higher than normal in men and mildly elevated in heterozygous women [35].

#### TTR

About 20% of T4 and 10-20% of T3 is bound in serum by transthyretin (TTR)/thyroxine binding prealbumin (TBPA), a homotetrameric protein synthetized in the liver. Over 120 different disease-associated mutations have been found in *TTR gene* (18q12.1), with variable manifestation. Most commonly they lead to amyloid deposition in the cardiac tissue and/or peripheral nerves, without affecting the status of thyroid hormones [15,36]. There is also a group of mutations that lead to a considerably increased affinity for iodothyronines (predominantly T4) and may result in euthyroid dysprealbuminemic hyperthyroxinemia with an elevated serum total T4, rT3 and fT4 index (TSH, fT4, T3 and fT3 unaffected) [37]. Dysprealbuminemic hyperthyroxinemia is transmitted in an autosomal dominant manner and accounts for approximately 2% of all euthyroid hyperthyroxinemia cases [38]. Mutations associated with an increased concentration of TTR do not cause euthyroid hyperthyroxinemia, as an observed augmentation in the protein level is not high enough (although in the course of some malignancies e.g. hepatocellular carcinoma, the TTR concentration may increase significantly and bring about the picture of euthyroid hyperthyroxinemia) [39]. Some of the *TTR* mutations lead to a decrease in protein blood concentration or a lesser affinity for iodothyronines, although without significant clinical or laboratory changes in thyroid hormones status.

#### Albumin

Familial dysalbuminemic hyperthyroxinemia (FDH) is another cause of inherited euthyroid hyperthyroxinemia. It results from *albumin gene* (4q13.3) mutations that lead to an increased affinity for fT4 and is inherited as an autosomal dominant disease. Although relatively rare, FDH is the most prevalent cause of inherited euthyroid hyperthyroxinemia in the Caucasian population (up to 12% of all cases, from 1:10,000 to 17:10,000) [40,41]. The following values are expected in laboratory tests: normal TSH (also after TRH stimulation) fT4, fT3, normal or slightly elevated T3, elevated T4, rT3 (in some cases) and an increased proportion of T4 bound to serum albumin (approximately four times higher than normally) [42]. The diagnostic problem may be falsely elevated fT4, as the laboratory techniques usually used are not accurate enough in that case, which may lead to unnecessary treatment [43]. In most cases, mutations at position 218 of the amino acid chain are present. However, another one was found in codon 66 (L66P) and leads to an increased affinity for fT3 and hypertriiodothyroninemia (FDH-T3) [44].

### Thyroid hormone resistance (RTH)

Thyroid hormones act primarily via nuclear receptors (TR): 1) TRα1 encoded by *THRA* (17q21.1) and 2) TRβ1 or TRβ2 encoded by *THRB* (3p24.2 ) (different expression pattern of both subtypes). T3 forms a complex with TR, and subsequently binds to the promoter region of the target genes (thyroid hormone response elements), acting as a transcription factor.

Mutations in TR genes lead to thyroid hormone resistance (RTH), disorder transmitted in the majority of cases in an autosomal dominant manner. Although no precise data are available, the prevalence is estimated to be 1:40,000 newborns [45]. In approximately 85-90% of RTH cases, the disease is caused by *THRB* mutations that lead to a decreased affinity for T3 or impaired interactions with TRβ cofactors. In these cases, elevated fT4 (often also fT3 and rT3) associated with normal or elevated (non-suppressed) TSH level is observed [46]. As a consequence of the variable manifestations observed among patients, RTH was formerly classified into generalized, isolated peripheral and isolated pituitary resistance. Often some evidence of both hyper- and hypothyroidism can be found in one patient (goiter, sinus tachycardia etc. may be associated with learning disabilities and delayed growth or bone age). In rare cases of homozygous *THRB* mutations, more severe symptoms are observed with coexisting deaf-mutism and color blindness [47].

Mutations in *THRA* were also described, which lead to different symptoms and thyroid function test results than in *THRB* defects. The symptoms of thyroid hormone deficiency are restricted to those tissues in which TRα predominates (central nervous system, myocardium, striated muscles, the gastrointestinal tract, cartilages and bones) [48]. Laboratory tests show a decreased fT4, very low rT3 and elevated fT3.

In a group of RTH patients (up to 15%), neither *THRB* nor *THRA gene* mutations were found (non TR-RTH), although phenotypically they may be indistinguishable from those with TRβ alterations. This subtype is also inherited in an autosomal dominant manner and expected to result from some TRβ cofactors mutations or defects in receptor regulation, although undefined to date [49].

Recently, the term ‘reduced sensitivity to thyroid hormones’ (RSTH) was introduced, which encompasses RTH and decreased responsiveness to thyroid hormones caused by iodothyronine transmembrane transport defect (mutations of MCT8 gene - *SLC16A2* in Xq13.2) or T4 to T3 deiodination defect (mutations of *SBP2* gene - *SECISBP2* in 9q22.2) [50-52].

### Rare causes of hyperthyroidism

Congenital hyperthyroidism, a generally rare disease (overt hyperthyroidism in 1:50,000 newborns), is most often transient and caused by the maternal thyroid stimulating antibodies in the course of Graves’ disease, subsequently transferred to fetal circulation (hyperthyroidism in 0.6-1% of offspring born to GD mothers) [53]. However, in some neonates there are no thyroid antibodies in serum, the mother’s disease is excluded and hyperthyroidism is persistent. This may suggest a condition called non-autoimmune hyperthyroidism (NAH). NAH develops as a result of gain-of-function germline mutation within the *TSH receptor gene (TSHR)*, leading to constitutive TSHR pathway activation. It can be divided into sporadic (SNAH) and familial (FNAH) with an autosomal dominant mode of inheritance [54,55]. To date, at least 21 different mutations of *TSHR* in FNAH and 12 in SNAH have been found. Similar mutations, but of a somatic type, can be found in toxic thyroid nodules [56]. Clinically, patients with NAH usually present with goiter and hyperthyroidism. Moreover, pharmacologic treatment may be ineffective (frequent relapses are observed) and total thyroidectomy or complete radioiodine ablation is usually necessary.

Hyperthyroidism is also observed in patients with McCune-Albright syndrome (MAS), as a result of the activating somatic mutation of the *GNAS gene*. As mentioned previously, GSα is a part of the TSHR signaling cascade and its constitutive activation is followed by a cAMP increase, which in turn results in thyrocyte hyperproliferation and iodothyronine excess. GSα is committed to extracellular signals transduction in different tissues as well. As *GNAS* mutations occur in MAS in the post-zygotic period and patients are mosaic, variable manifestations are observed. The classical triad of symptoms include bone fibrous dysplasia, café-au-lait spots and hyperfunctional endocrinopathies (most frequently peripheral precocious puberty, but also hyperthyroidism, hypercortisolism, hypophyseal hyperfunction and kidney phosphate wasting). MAS prevalence range from 1:100,000 to 1:1,000,000 [57], with functional or morphological changes in the thyroid in approximately 31% of cases [58].

Symptoms of hyperthyroidism may also be observed, albeit rarely, in the course of struma ovari (5-15% of cases), differentiated thyroid cancer (usually methastatic) and an gestational trophoblastic disease [59-61]. Furthermore, central (secondary) hyperthyroidism due to TSH secreting pituitary adenoma can be classified as a rare disease as it accounts for only about 1% of all pituitary adenomas (prevalence of 1:1,000,000) [62].

### Rare thyroid inflammatory diseases

Thyroid gland tuberculosis is very rarely observed in the course of generalized disease (even in regions with a relatively high incidence of tuberculosis) and much less frequently as a change isolated or primarily localized in the thyroid. Analyses of surgically removed glands or fine needle aspiration biopsy (FNAB) materials showed a prevalence of 0.1-0.6% [63,64]. Thyroid tuberculosis may present as a single nodule, multinodular goiter or diffused swelling, it may be found as a cold abscess or rarely an acute abscess [65]. Some patients remain symptomless, while others develop dyspnea, dysphagia, hoarseness, pain or tenderness. Typically, the hormonal status of the thyroid gland is unaffected, although hyperthyroidism may occur in some cases, as a result of excessive release of thyroid hormones from damaged tissue. Hypothyroidism, which is extremely rarely observed, may be caused by extensive tissue destruction [66]. To establish the diagnosis, ultrasound-guided FNAB should be performed with Ziehl Neelsen staining, culture and cytological examination (caseating granulomas with epithelioid cells and Langhans giant cells).

Sarcoidosis - a noncaseating granulomatous disorder, may rarely affect the thyroid gland in the course of generalized disease. Usually, extrathyroidal manifestation of the disease precedes the diagnosis of thyroid involvement. Some rare cases of sarcoidosis limited to the thyroid gland can be also found in the literature [67]. In post-mortem studies of patients with previously found systemic sarcoidosis, the thyroid gland was affected in up to 4.5% [68]. Thyroid sarcoidosis usually presents as a progressive painless enlargement of the gland with unaffected hormonal status, although different manifestations are possible (hyperthyroidism, hypothyroidism, acute thyroiditis, multinodular goiter or solitary thyroid nodule, it may sometimes be painful). Cases of hyperthyroidism resistant to both pharmacological and radioiodine treatment were observed in the course of thyroid sarcoidosis, and in these cases surgical removal of the gland is required. Studies also suggest an increased prevalence of high antithyroid antibodies concentration and thyroid autoimmune diseases associated with sarcoidosis, regardless of thyroid gland involvement (an ATD frequency in patients with sarcoidosis 1.9-16,6%) [69].

## Conclusions

Most of the rare thyroid diseases presented in this paper have a genetic origin, among them monogenic disorders can be found. Progress in our understanding of their etiology and pathogenesis has been observed in the past years, what also shed new light on how the normal thyroid gland functions and the role of different proteins in this process, although there are still some questions that need answers. Secondly, rare thyroid diseases may be a challenging problem for clinicians because of their rarity and variability in manifestations. They should be kept in mind as a differential diagnosis of other diseases more commonly seen in clinical practice. A correct and undelayed diagnosis is especially important in the case of congenital thyroid function disruptions, as untreated, they may lead to serious consequences.
